# Unfavourable outcome of deep brain stimulation in a Tourette patient with severe comorbidity

**DOI:** 10.1007/s00787-012-0285-6

**Published:** 2012-05-24

**Authors:** A. Duits, L. Ackermans, D. Cath, V. Visser-Vandewalle

**Affiliations:** 1 Department of Psychiatry and Psychology, Maastricht University Medical Centre, PO Box 5800, 6202 AZ Maastricht, The Netherlands; 2 Department of Neurosurgery, Maastricht University Medical Centre, Maastricht, The Netherlands; 3 Maastricht Institute for Neuromodulative Development (MIND), Maastricht, The Netherlands; 4 School of Mental Health and Neuroscience (MHeNS), Maastricht University, Maastricht, The Netherlands; 5 Altrecht Academic Anxiety Outpatient Services, Department of Clinical Psychology, Utrecht University, Utrecht, The Netherlands; 6 European Graduate School of Neuroscience (EURON), Maastricht, The Netherlands

Dear Editor(s),

In 1999, deep brain stimulation (DBS) was introduced as a new therapeutic approach for intractable Tourette syndrome (TS) [1]. Currently, it is still considered to be an experimental option with limited controlled data to support its effect [2, 3]. Both single and multiple brain areas have been targeted to deal with tics as well as comorbid symptoms of TS, including attention deficit hyperactivity disorder (ADHD), obsessive–compulsive disorder (OCD) and behaviour (OCb), self-injurious behaviour (SIB), depression and anxiety. These associated comorbidities are present in up to 90 % of clinically referred TS patients and have a significant impact on quality of life [2]. So far, guidelines to select patients [4, 5] for DBS were mainly focused on tics. Also in the recently revised clinical guidelines by the European Society for the Study of Tourette Syndrome [2], the issue comorbidity in patient selection is relatively underaddressed.

In 2010, we have completed a double-blind trial stimulating the centromedian nucleus–substantia periventricularis nucleus–ventro-oralis internus (Cm–Spv–Voi) cross-point of the thalamus [3]. Here, we report on the outcome of DBS in an institutionalized patient with intractable TS and severe comorbidity. Although she was younger than 25 years, she was included in the trial and underwent surgery for clinical urgency. Postoperatively, she could not be randomised within the period of follow-up because of unexplained complications. In this letter, we discuss the nature of the complications and the implications for patient selection.

The 20-year-old female patient was born with a mild spastic diplegia due to complications during pregnancy. The diagnosis of TS was made at the age of 10 years. Motor and vocal tics included eye blinking, licking, cheek biting, tapping with her right foot, coughing, barking, uttering sounds and coprolalia. Associated comorbidities included depression, pervasive developmental disorder not otherwise specified (PDD-NOS) and compulsions (e.g., forced touching of furniture and a specific form of trichotillomania). In 2005, she developed severe SIB, including repetitive hitting her head and arm (she had to wear a helmet and a body protector), poking sharp objects into her body and scratching until bleeding. Numerous pharmacological treatments had been administrated and discontinued, due to the side effects or lack of response. Behavioural therapy and electroconvulsive therapy were even leading to new tics. In November 2006, she underwent surgery for thalamic DBS. See Ackermans and colleagues [3] for the surgical details.

Three weeks after surgery, while adjusting the parameters, the patient showed an abrupt onset of severe hypertonia in her left leg. In the following days, this extended to the right leg and both arms, followed by involuntary movements of the left arm, and on one occasion rotating movements of both arms above the head. The symptoms did not disappear when switching off the stimulator. Either with or without stimulation, the hypertonia was present but inconsistent over time and could not be classified within the phenomenology of organic movement disorders such as myoclonus, dystonia or spasticity. During the first month after surgery, she was bedridden and developed an opisthotonus. Rehabilitation with a cognitive behavioural approach was not successful. In May 2007 she developed unexplained disturbances of consciousness, followed by periods of mutism. To exclude a side-effect of stimulation, double-blind stimulation was performed at 19 months after surgery according to the procedure of the trial [3]. Unblinding the conditions showed an increase in tics (see Table 1), but a relief of hypertonia while the stimulator was switched on, and visa versa. For the patient, the results were completely unexpected and she decided to have the stimulator switched off. From that time on she showed alternating hypertonia in legs and arms, but relatively few motor and vocal tics. Later, she developed complaints of impaired swallowing, nausea and she could not bear food anymore. Diagnostics (e.g., gastroscopy) did not result in an organic explanation and psychiatric consultation did not reveal a depressive state. Hypodermoclysis could not prevent dehydration and she refrained from admission to a hospital for intravenous infusion. In November 2009, she passed away in a nursing home. For personal reasons no autopsy was performed.

**Table 1:** Pre- and post-operative assessment of tics and comorbidities

	Before surgery^a^	Stimulation off^b^	Stimulation on^c^
Tic severity (YGTSS total)	42	12	39
Vocal tics (YGTSS vocal)	17	0	14
Motor tics (YGTSS motor)	25	12	25
OCD (Y-BOCS)	20	8	7
SIB (VAS)	5.7	0	8.9
ADHD (CAARS)	58	42	45
Autism (PDD-SQ)	136	127	124
Depression (BDI-II)	12	20	15
Anxiety (BAI)	32	41	44

*YGTSS* Yale Global Tic Severity Scale, *OCD* obsessive compulsive disorder, *Y-BOCS* Yale-Brown Obsessive Compulsive Scale, *SIB* self injurious behaviour, *VAS* Visual Analogue Scale, *ADHD* attention deficit hyperactivity disorder, *CAARS* Conners Adult ADHD Rating Scale, *PDD-SQ* Pervasive Developmental Disorder-Self Questionnaire, *BDI-II* Beck Depression Inventory (2nd edn.), *BAI* Beck Anxiety Inventory

^a^Pre-operative medication included duloxetin (30 mg/day), alprazolam (0.50 mg 4 times a day), baclofen (20 mg thrice a day) and pergolide (0.05 mg/day)

^b^22 months after surgery, at the end of the first randomization period of 3 months with stimulation ‘off’

^c^23 months after surgery, at the end of the second randomization period of only 6 weeks with stimulation ‘on’ and the following stimulation parameters: 5 mV, 60 μs and 110 Hz (contact 1−/2+, 5−/6+) bilaterally

Considering the criteria of Fahn and Williams [6], the abrupt onset of the hypertonia, its inconsistency and incongruency with organic disorders, the bizarre movements and the concomitant somatizations (loss of consciousness, mutism and later swallowing disturbances) suggest a psychogenic movement disorder. The surgical procedure or stimulation may have caused a dysbalance in the limbic and associative cortico-basal ganglia-thalamocortical loops [7], leading to psychiatric symptoms. Perhaps, the symptoms could be seen as disinhibition or progression of a preoperatively unidentified somatoform disorder. Reviewing the patient’s medical history, it is noteworthy that she frequently suffered from abdominal distress, headaches and unexplained urine retention (related to decreased relaxation of pelvic muscles).

Finally, the decrease in tic severity, which possibly served as a coping mechanism to daily demands prior to surgery, might have interfered with the results of DBS.

Although it is difficult to draw final conclusions, we believe that the course of this case illustrates the potential contra-indication for DBS in TS patients with severe comorbidity. While TS is a heterogeneous entity [8], the combination of associated comorbidities with prominent and severe SIB may raise questions about the diagnosis. Whereas the diagnosis of TS had been approved by several experts in TS prior to surgery, our patient might also have suffered from an additional somatoform disorder. A somatoform disorder can be treatment-resistent as well, but contrary to TS, DBS is not indicated and might even worsen the clinical picture. Apart from the psychiatric comorbidity, our patient also suffered from a pre-existent neurological disorder, spastic diplegia, which in itself may constitute a risk for DBS.

In conclusion, before deciding upon DBS, we would like to emphasize that experts in TS should confirm the diagnosis and that patients should be thoroughly screened for somatoform disorder, including psychogenic movement disorder. Patients should be excluded from DBS in the presence of symptoms. Overall, clinicians should be alert on comorbid psychiatric symptoms beyond the well-known associated comorbidities such as OCD and ADHD. Serious preoperative attempts to treat psychiatric comorbidities are warranted even in case of apparent clinical urgency to perform surgery.
